# Mapping Shape to Visuomotor Mapping: Learning and Generalisation of Sensorimotor Behaviour Based on Contextual Information

**DOI:** 10.1371/journal.pcbi.1004172

**Published:** 2015-03-27

**Authors:** Loes C. J. van Dam, Marc O. Ernst

**Affiliations:** 1 Department of Cognitive Neuroscience, Universität Bielefeld, Bielefeld, Germany; 2 Cognitive Interaction Technology (CITEC) Center of Excellence, Universität Bielefeld, Bielefeld, Germany; 3 Bernstein Center for Computational Neuroscience, Tübingen, Germany; University College London, United Kingdom

## Abstract

Humans can learn and store multiple visuomotor mappings (dual-adaptation) when feedback for each is provided alternately. Moreover, learned context cues associated with each mapping can be used to switch between the stored mappings. However, little is known about the associative learning between cue and required visuomotor mapping, and how learning generalises to novel but similar conditions. To investigate these questions, participants performed a rapid target-pointing task while we manipulated the offset between visual feedback and movement end-points. The visual feedback was presented with horizontal offsets of different amounts, dependent on the targets shape. Participants thus needed to use different visuomotor mappings between target location and required motor response depending on the target shape in order to “hit” it. The target shapes were taken from a continuous set of shapes, morphed between spiky and circular shapes. After training we tested participants performance, without feedback, on different target shapes that had not been learned previously. We compared two hypotheses. First, we hypothesised that participants could (explicitly) extract the linear relationship between target shape and visuomotor mapping and generalise accordingly. Second, using previous findings of visuomotor learning, we developed a (implicit) Bayesian learning model that predicts generalisation that is more consistent with categorisation (i.e. use one mapping or the other). The experimental results show that, although learning the associations requires explicit awareness of the cues’ role, participants apply the mapping corresponding to the trained shape that is most similar to the current one, consistent with the Bayesian learning model. Furthermore, the Bayesian learning model predicts that learning should slow down with increased numbers of training pairs, which was confirmed by the present results. In short, we found a good correspondence between the Bayesian learning model and the empirical results indicating that this model poses a possible mechanism for simultaneously learning multiple visuomotor mappings.

## Author Summary

When interacting with the world, for instance, when pointing to targets, humans generally make use of previous experiences to determine the best course of action. For example, based on the context of wearing corrective glasses or taking them off, we instantly adjust our behaviour for the geometric distortions the glasses induce. How do we learn such context-dependent distortions and how does such learning generalise?

We translated this question into a target-pointing task in which different target shapes were each associated with a different distortion. That is, participants needed to aim right-of-target to “hit spiky shapes and left-of-target for round ones. In this way we exposed participants to a linear relationship between target shape and required motor response. After training, performance was measured for shape-morphs along the spiky-round axis that were not explicitly part of the training set (generalisation). We found that participants did not use the trained linear relationship, but rather weighed the learned shape-contexts according to their similarity to the current test-shape. Furthermore, learning substantially slowed down with increased numbers of training shapes. We developed a Bayesian learning model capturing these results. The model demonstrates that participants learn the set of separate shape-response pairs rather than their relationship.

## Introduction

When interacting with the world around us, we largely depend on prior knowledge about the structure of the world and the relationships between the sensory signals resulting from it in order to choose the appropriate action to achieve our goal. This prior knowledge is not necessarily fixed. Rather, through experience we can learn new perceptual [1–3] and sensorimotor relationships (for reviews see e.g. [4–6]). For instance, when wearing magnifying glasses, the way in which visual positions relate to actual positions in the real world will change, and this changed relationship even depends on the eccentricity of the visual location relative to our head. We perceive objects to our left more to the left and objects to the right more to the right. Due to this changed relationship we may initially experience some problems when trying to look at or reach for any object, but we easily adapt our behaviour to this changed relationship in a relatively short amount of time (see e.g. [7, 8]). That is, we can easily learn the association between visual location and required movement to successfully aim for a target.

This type of associative learning is quite general and even extents to contextual cues that are not necessarily directly task relevant. For instance, the colour of a target for a pointing task normally is irrelevant for how we perform the pointing movement. However, we can learn that we have to point a certain extent to the right for blue targets and to the left for red ones in order to “hit” them [9]. In other words we can learn the association between the target colours, i.e. the contextual cues, and the leftward and rightward mappings. When, after training, either of these target colours is presented, we immediately switch to the correct mapping without the need for feedback to correct our movements.

That we can learn such associations for different visuomotor mappings has been shown for a number of contextual cues, such as simply wearing prism glasses or not [10–13]; the presence or absence of an auditory tone [14]; head tilt towards left or right shoulder [15]; different prism shifts for different visual locations using split-field prisms [8, 16]; different arm-loads due to the presence or absence of a wrist weight bracelet [17] and different target colours [9, 18]. It is important to note, however, that in most of these studies the role of the learned associations was investigated for only two discrete association-stimulus/visuomotor-mapping pairings. That is, in many of these studies, the context cue could often only have one of two discrete states: the presence or absence of the cue. In this case, testing conditions cannot go beyond the specifically trained pairings to see how the trained pairings generalise to novel conditions (a cue cannot be half present). Furthermore, using such discrete cues, the effect on the number of pairings on the learning rate was never tested. However, many cues, like the location of targets when learning the distortions for a single pair of glasses, can vary in a continuous manner. The question arises how learning for a discrete number of trained pairings affects the learning rate and generalises to novel conditions on the same continuous cue scale.

In the example of the magnifying glasses, one could for instance simply learn, from a few example locations, a new linear rule between the visual location of a target and the behavioural shift needed to deal with the magnification. This linear rule would then also be applied to conditions that were not explicitly trained. One could imagine that learning such a rule might in principle be faster than learning the mapping for each and every location separately. However, the structure of a new relationship that needs to be learned can in principle be quite complex. For instance, around the edges of the magnifying glasses large non-linearities in the structure arise. In this respect, applying a general linear rule would not always be beneficial and it might even be better not to generalise beyond the trained conditions at all. To learn the full complexity of the structure we would then have to experience many samples along the full scale of possibilities, which would slow down the learning process. This simple example demonstrates that there are many possibilities to generalise the learned associations, and each possibility has its own costs and benefits.

The picture that arises from the existing literature on generalisation in visuomotor tasks is also not very consistent. Bedford [7] was one of the first to investigate generalisation after training with differential prism shifts for one, two, or three separate target locations. She found that participants did seem to adopt the simplest linear rule when interpolating to new test target locations even when the relationship between the three training conditions was non-linear. Other studies that used only one visual location to train a new visuomotor mapping found that generalisation was more or less restricted to the trained location with a steady decrease in learning effect the further away the test location was from the trained location [19–21]. Very limited generalisation was also found for different starting positions of the movements [22], different movement speeds [23], trained left or right hand [13, 23] and type of movement, i.e. overhand or underhand throwing [13].

The problem with many of these studies, however, is that the cues for the separate mappings were directly task relevant (e.g. target location and movement speed directly relate to the movement requirements), and in some cases even discrete (e.g. hand used, type of throw). Using task relevant cues poses a problem in the sense that learned associations between the used cues and visuomotor behaviour will likely pre-exist before training starts, simply through experience in normal life. For instance, to interact with objects we often use our left and right hand independently, and from this experience a separate mapping for each hand could already exist with which generalisation of learning for one hand only would have to compete. Such pre-existing associations would, of course, also naturally interfere with any experimental approach. This is one possible reason for the limited extent of generalisation found in some cases (e.g. [19–21]). Furthermore, as noted above, discrete cues, such as for instance the throwing hand, cannot provide information about possible generalisation rules on a continuous scale of associative cues.

**Fig 1:**
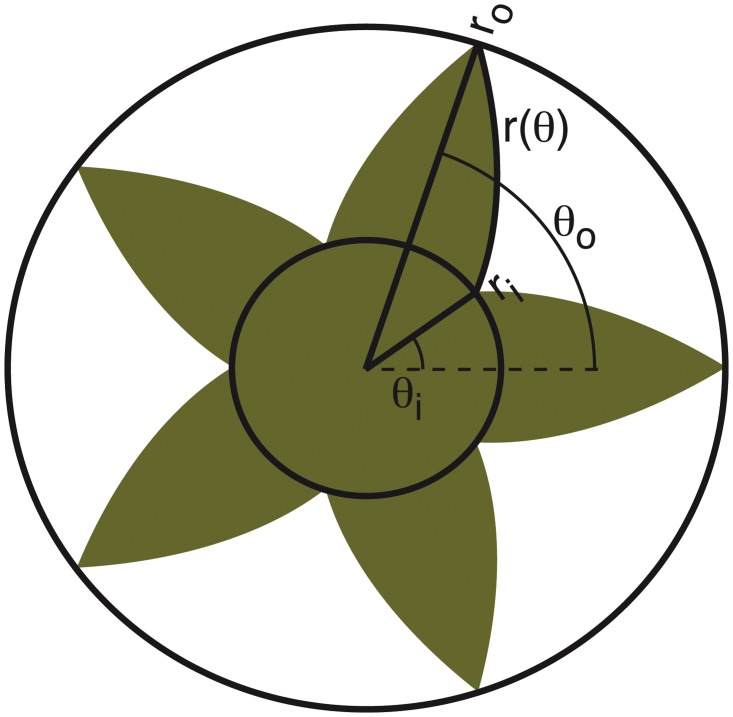
Shape definition parameters. Each shape has a separate morph factor which is defined by the ratio between the inner and outer radii of the shape $\rho =\frac{r_{i}}{r_{o}}$. The bulgy spikes are obtained by making the radius *r* dependent on the angle *θ* within the shape via: $r(\theta )=r_{i}e^{\left(\frac{\theta -\theta_{i}}{\theta_{o}-\theta_{i}}\right)\ln\left(\frac{r_{i}}{r_{o}}\right)}$. The inner and outer radii for each *ρ* were chosen such that the surface area matched that of a circular disk with radius *R* = 1.3 deg (12.5 mm). The two radii were defined as: $r_{o}=R\sqrt{\frac{2\ln\left(\rho \right)}{\rho^{2}-1}}$ for (0 < *ρ* < 1), *r*_*o*_ = *R* for (*ρ* = 1) and *r*_*i*_ = *ρ* *r*_*o*_. For a full description of the shape definition see S1 Text.

In the present study we circumvented these problems by investigating how learning and generalisation occurs for associations with a task-irrelevant property of the target in a rapid pointing task. Here, we chose target shape as the contextual cue that ranged from circular to spiky on a continuous scale (see Fig. 1, cf 3). That is, each shape along the scale could be identified by a single continuous shape parameter, the morph factor *ρ* (see Fig. 1). Continuously with the shape cue, we varied the visuomotor mapping to be learned, that is, the visuomotor response to reach the target location. For instance, participants had to point more to the right in order to hit spiky target shapes and more to the left to hit the round ones. We trained participants on only a subset of shape/visuomotor-mapping pairs and after training we tested for generalisation to other target shapes along the shape scale. The relationship between shape and visuomotor mappings underlying this training was always linear. If participants would succeed in learning this underlying linear relationship, generalisation can be expected to occur by inter- and extrapolating linearly from the shape-mapping training pairs. In different experiments we varied the number of training pairs (target shape/mapping pairings) to investigate differences due to the amount of information available for extracting the linear rule for generalisation to novel shapes as well as how the number of training pairs affects learning rates. That is, more training pairs should provide more information about the linear relationship and thus this should lead to an increased chance of identifying the generalisation rule without much loss in terms of the time needed for learning it.

The prediction for linear generalisation derived above represents the simplest rule that connects all the trained shape/mapping associations. However, this prediction only specifies how we should generalise once we have learned this linear association. It does not specify how the learning itself occurs and thus, for instance, does not make quantitative predictions with respect to the learning rate. In other words, it would be useful to understand the possible learning mechanism that leads to the generalisation in the end, instead of hypothesising only the generalisation pattern. For this purpose, we developed a Bayesian learning model in which we combine ideas from optimal motor control and Mixture-of-Experts generalisation. In the Bayesian learning model, a new mapping is learned separately for each target shape. The optimal model for learning such a single mapping is the Kalman filter (Equation 1–4 in Bayesian Learning Model) and it has been shown that in many ways human motor learning resembles the behaviour of such an optimal model (see e.g. [24–26]). Thus, in our model each target shape is associated to one such optimal learner/Kalman filter. During and after training, generalisation is achieved by averaging these mappings with a generalisation gradient around the currently presented target shape. This generalisation process in fact is a weighted average across the optimal learners and is very similar to the Mixture-of-Experts Model that has previously been used to describe the generalisation from associations between the starting position of the movement and the visuomotor mapping required to reach the target [27]. In our case, each Kalman filter represents such an “expert” for the shape associated with it. It follows that the associative learning process investigated here could very well be described by a Mixture-of-Kalman-Filters where each shape along the continuous shape scale is coupled to its own learning mechanism (see Bayesian Learning Model in Materials and Methods). Note, this model does not learn the underlying shape/mapping relationship, but learns the shape/mapping relationship only locally with a generalisation gradient around it. Furthermore, the Bayesian learning model predicts that the learning rate depends on the combination of shape-mapping pairs used during training. When multiple pairs need to be learned simultaneously, these will interfere with each other due to the generalisation of local learning. The model therefore predicts a slow down of the overall learning rate with an increased number of shape-mapping pairs to be learned. Simulations from this model were compared to the empirical results as an alternative hypothesis to the linear generalisation rule. We found that the Mixture-of-Kalman-Filters Model predicts the pattern of generalisation and also the pattern of observed learning rates quite well and better than the simple linear generalisation prediction.

## Results

### Experiment 1

**Fig 2:**
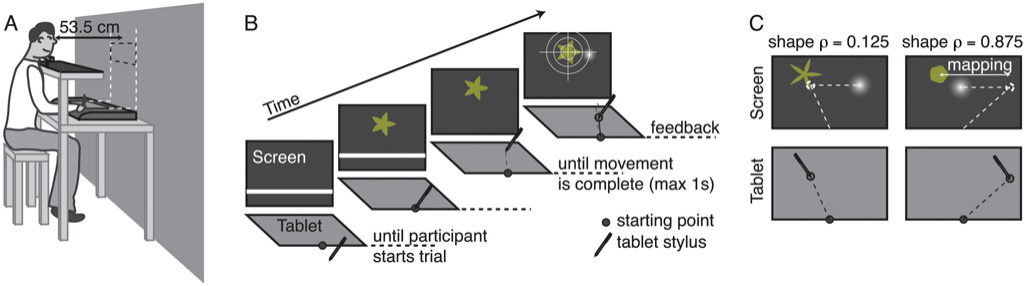
Setup and procedure. A) Experimental setup. B) time course of one single trial. Participants initiated the trial by tapping at the starting point after which a target was shown. The trial ended when the participant tapped on the tablet again at the end of their pointing movement, upon which the visual feedback was shown. C) Use of cued mappings. In Experiment 1, two mappings were trained. The -75 mm mapping (i.e. in order to hit the target participants had to point 75 mm to the left of the target, corresponding to a 75 mm rightward shift of the feedback relative to the actual pointing location) was cued by a relatively spiky shape (*ρ* = 0.125). The second mapping of +75 mm was cued by an almost circular shape (*ρ* = 0.875). The left column shows an example in which the participant had not yet learned to use the cue. In the right example the participant has already learned the cued mapping and points to the right of the actual target location.

Participants performed a target-pointing task in which we manipulated the spatial mapping of the visual feedback that participants received upon completion of their pointing movements. This corresponds to changes occurring in the environment affecting the visuomotor mapping, such as when wearing optical glasses. We generated different target shapes using a morphing method. Shapes ranged from very spiky to completely round. Each target shape was associated with a particular visuomotor mapping in such a way that there was a linear relationship between them. In the first experiment, participants were trained on two specific target shape/mapping contingencies along this continuous scale. One training combination was a shape with *ρ* = 0.125 (a relatively spiky shape) and a mapping of −75 mm (i.e., visual feedback was shifted by 75 mm to the right with respect to the actual pointing location, such that the participant would have to point 75 mm to the left of the target in order to directly hit it, i.e. to get the feedback on target, cf. Fig. 2C); the other training combination was shape *ρ* = 0.875 (almost circular) and a mapping of +75 mm. Afterwards, using catch trials for which the task-relevant visual feedback was eliminated, we tested participants’ pointing performance to target shapes that had not been used explicitly during training. In this first experiment, we specifically tested participants’ performance on different target shape morphs that could either be in-between the two training shapes along the shape morph axis (interpolation) or that could come from further outside (extrapolation). Participants were naive to the purpose of the experiment and thus also to the meaning of the target shapes. However, to motivate participants to use all the cues available (both feedback and target shapes) they received a score after each trial dependent on the absolute visual error between target location and feedback location (100 points if the absolute pointing error was below 1 cm; 50 points when between 1 and 2 cm; 25 between 2 and 3 cm and 0 otherwise). After the experiment we debriefed participants using a questionnaire where they answered whether they had noticed the different shapes and their contingencies with the required pointing behaviour.

We first analysed whether the participants had learned the shape-mapping contingencies for the two shapes used during training. To do so, we examined whether there were significant differences in the pointing behaviour for the different shapes (including generalisation shapes) at the end of training for each individual participant. The test performed was a one-way ANOVA with shape as the only factor. If there were significant differences depending on the shape, we concluded that the participant must at least partially have learned the shape-mapping contingencies. Otherwise behaviour should not have differed for the different shapes. Using this criterion it turns out that 7 out of the 12 participants showed significant learning of the shape-mapping contingencies. Interestingly, the seven participants who learned the contingencies were all also participants who had consciously realised the meaning of the different shapes. Only one additional participant who realised the meaning of the shapes with respect to the required mappings failed to adjust pointing behaviour accordingly. This participant continued to point to where to target was without adjusting for the error in the feedback. Participants who had not noticed the shape-mapping contingency also did not learn to adjust their behaviour differentially corresponding to the target shape. Thus, from these results it seems that in order to learn the different mappings, awareness of the meaning of the shape with respect to the mapping is necessary.

**Fig 3:**
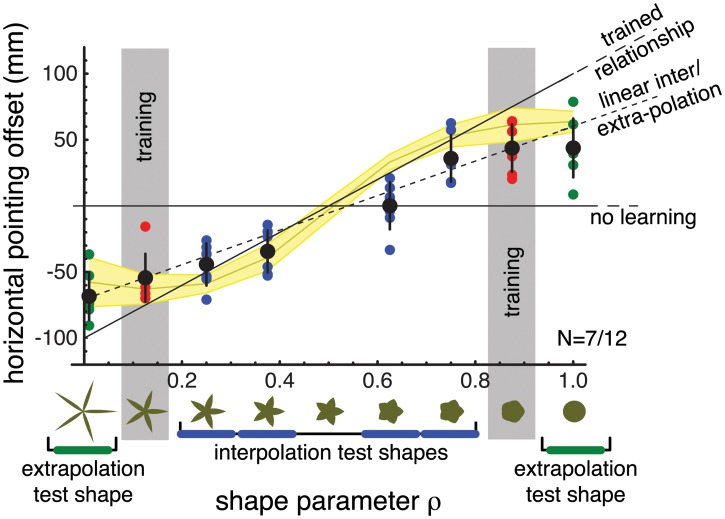
Generalisation results of Experiment 1. Results are shown for participants that learned the cued mappings (7 out of 12 participants). The x-axis indicates the training and test shape morph factors. The y-axis indicates the mapping that participants applied for each shape. The continuous horizontal and tilted black lines indicate the no-learning prediction and the trained linear relationship, respectively. The black dashed line indicates the prediction for linear inter/extrapolation based on the linear fits of the training results. Coloured disks indicate single participant results for the corresponding test shape (red, blue and green disks represent trained shapes, interpolation shapes and extrapolation shapes, respectively). Black circles and error bars indicate the mean and standard deviation across participants. The yellow line and shaded area show the Mixture-of-Kalman-Filters Model mean and standard deviation across participants. The results indicate that the learning for 2 trained shape-mappings pairs transfers to the other shapes on the same morph scale. Both linear generalisation and the Mixture-of-Kalman-Filters Model seem to capture the observed pattern of generalisation.

For the participants that learned the shape-mapping contingencies we further investigated if and in what manner learning for the two training shapes transfers to other shapes along the shape-scale. The participants who had not learned the contingencies were excluded from this analysis since it does not make sense to test for transfer if they did not learn the contingencies to begin with. The results for the participants that learned the shape-mapping contingencies in Experiment 1 are shown in Fig. 3. Here, the mapping employed behaviourally is depicted as a function of the shape parameter. The results for the two training conditions are indicated by grey bars.

What seems evident from the data is that there is some form of transfer of learning from the trained shape-mapping contingencies to the other shapes along the scale. If there had been no transfer, the results for both the inter- and extrapolation shapes should be close to 0. For generalisation shapes (blue and green points) the results seem to follow more or less a linear trend. We tested whether the linear hypothesis could be rejected by performing an ANOVA on the linear regression residuals for each participant. If the learning and transfer of learning occurs on a linear scale there should not be a difference in the residuals for the different shapes used in the experiment. As it turns out (after Bonferroni correction), the results for all participants but one (n = 6) conformed with this linear hypothesis. That is, the hypothesis that transfer of learning likely occurs in a linear fashion cannot be rejected. However, the test shapes in this experiment were chosen mostly from shapes that were intermediate with respect to the trained shapes (interpolation shapes). For the extrapolation shapes the trend is not so clear, given the small number of shapes (i.e., 2) used to test for extrapolation. Also results seem to differ on both sides of the shape range: extrapolation to the spiky shape seems increased from the adjacent trained shape but extrapolation to the circle, though quite different from zero, does not seem to be linearly increased from the adjacent training shape. So these results do not allow for a firm conclusion.

It is important to note that also the Mixture-of-Kalman-Filters performs quite well in predicting the generalisation behaviour although it sometimes slightly overestimates the learning extent for the training shapes (difference between yellow line and red dots) leading to significant differences in the residuals for 3 out of 7 participants after Bonferroni correction. In the Mixture-of-Kalman-Filters generalisation comes about locally by the learning for one shape also influencing the mapping for neighbouring shapes (Equation 5 in Materials and Methods). In the model, the gradient for this local generalisation is directly linked to the discrimination threshold between the different shapes (see Bayesian Learning Model in Materials and Methods and Supporting S2 Text) and therefore was not a free parameter. When comparing *R* ^2^ for linear generalisation and the Mixture-of-Kalman-Filters, the latter performs slighty better (comparisons of *R* ^2^ in a signed-rank test leads to p = 0.03; mean $R_{\text{MKF}}^{2}$ = 0.77; mean $R_{\text{Lin}}^{2}$ = 0.60) in spite of having one free parameter less per participant. Given that both hypotheses match the results quite well it would be premature to conclude which of the two hypotheses should be preferred.

### Experiment 2

To further investigate the transfer of learning with a focus on extrapolation we performed more experiments in which the training conditions were shifted towards shapes that were more to the centre of the shape-scale. Furthermore, we also investigated the influence of the number of different training shapes along the scale. The prediction is that the more shape-mapping pairs are provided during training, the more evidence there is for a linear correspondence between them. Thus, it can be expected that interpolation and especially extrapolation becomes more linear with increasing numbers of training pairs if the linear relationship was learned.

We tested two different training conditions, each using a new group of participants who had not participated in Experiment 1. In the first condition two training shape-mapping contingencies were again used. However, now the two training shapes were picked from a more central position along the shape-scale. The shape-mapping pairs that were trained were the combinations: *ρ* = 0.25 and a mapping of −50 mm and *ρ* = 0.75 combined with a mapping of +50 mm. In the second condition of Experiment 2 we extended the number of shape-mapping pairs to five: (*ρ* = 0.25, mapping = −50mm), (*ρ* = 0.375, mapping = −25mm), (*ρ* = 0.50, mapping = 0mm), (*ρ* = 0.625, mapping = 25mm) and (*ρ* = 0.75, mapping = 50mm). These five pairs span the same range as in the two-pair condition but provide more evidence for a linear relationship between shape and mapping. Therefore, we expect the extrapolation in the 5-Pair Condition to be more linear than in the 2-Pair condition, in case this linear relationship would have been learned. On the other hand, if the Mixture-of-Kalman-Filters Model were correct, we would predict a similar generalisation pattern for both conditions: the applied mapping for extrapolation shapes stays more or less constant compared to the nearest training pair.

From Experiment 1 we know that awareness of the role of the shapes as a cue for the associated mapping seems necessary for learning to occur. Since we were interested in the transfer of learning we wanted to increase the chance for learning. Therefore, in Experiment 2 we informed participants about the fact that different shapes would need different pointing behaviours. However, the participants were not informed about the particular contingencies between the shape and mapping which they had to deduce themselves. Thus, participants were also not informed about the linear relationship between the shape and mapping.

**Fig 4:**
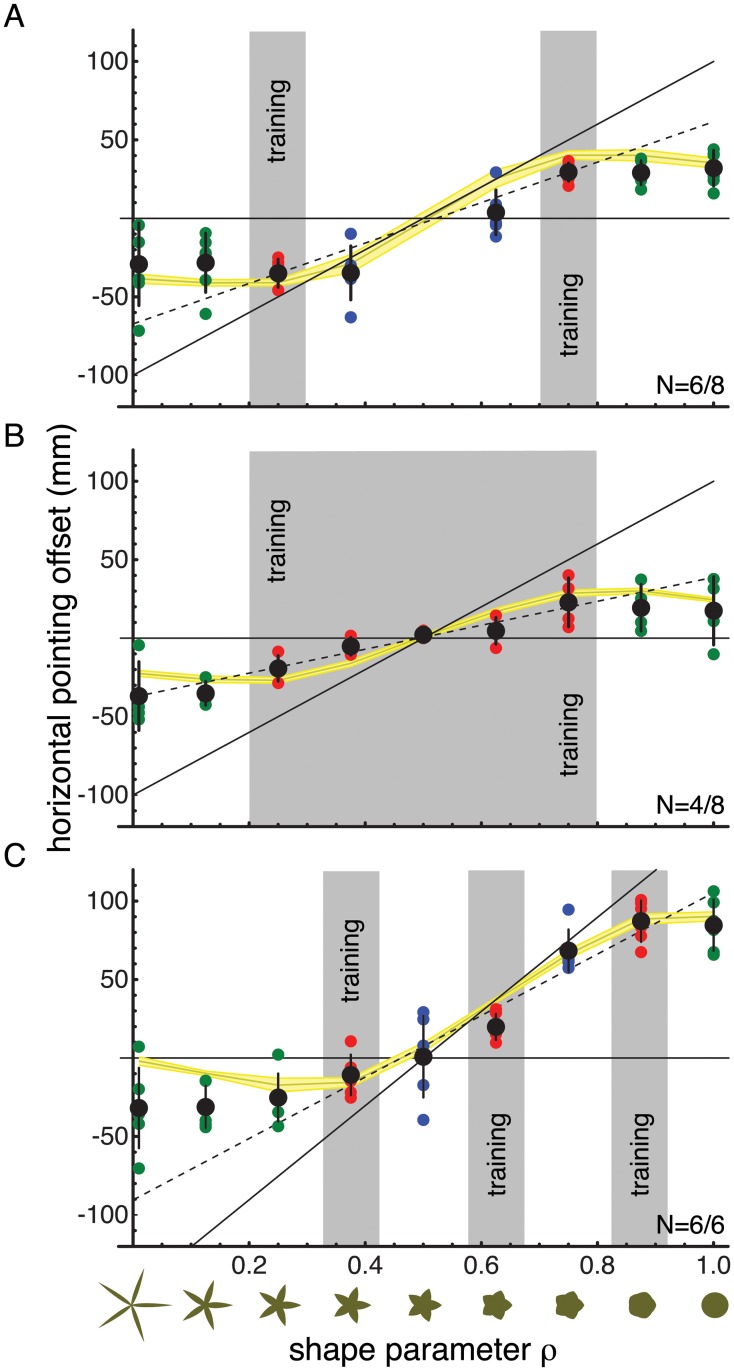
Generalisation results for Experiments 2 and 3. A) Results for the 2-Pair Condition of Experiment 2. B) Results for the 5-Pair Condition of Experiment 2. C) Results for Experiment 3 in which 3 training pairs were used. In each graph the numbers in the lower right corner indicate how many participants learned and the total number of participants for that condition. Axes and colour coding are the same as for Fig. 3. The results indicate that extrapolation of learning does not occur in a linear manner. The Mixture-of-Kalman-Filters performs better at capturing the generalisation results.

The results of Experiment 2 are shown in Fig. 4A for the 2-Pair Condition and in Fig. 4B for the 5-Pair Condition. Notably, and maybe counter-intuitively if people were able to learn the linear relationship, learning the shape-mapping contingencies seemed to be much harder in the 5-Pair Condition than in the 2-Pair Condition. Out of the 8 participants for the 5-Pair Condition only four actually showed learning compared to six participants in the 2-Pair Condition. Furthermore, the extent to which the participant learned the contingencies was less for the 5-Pair Condition than the 2-Pair Condition. If we consider the training shapes only, the behavioural slope between mapping and shape after training was only 38% of the actually trained slope for the 5-Pair Condition compared to 64% for the 2-Pair Condition. This difference in the amount of learning between the two conditions was significant (t-test, p = 0.03) even with our relatively small sample size. Further, note that for this analysis the participants that failed to learn were already removed. Thus, this effect in the amount of learning cannot be due to the fact that fewer participants learned in the 5-Pair Condition.

It is however important to note, that since we kept the overall number of training trials in the separate conditions the same, each shape-mapping training pair in the 5-Pair Condition received fewer examples than each pair in the 2-Pair Condition. To get a more equivalent comparison for the amount of learning with respect to the number of training trials per pair, we compared the learning extent of the 2-Pair Condition after two training blocks of trials with the learning extent of the 5-Pair Condition after five such blocks. This comparison ensures that participants have seen each training pair equally often (ca. 180 times). It turns out that even for this comparison, when the number of examples per pair are equalised across conditions, the learning extent in the 2-Pair Condition is significantly larger than the 5-Pair Condition (t-test, p = 0.02). Thus, this provides further evidence that increasing the number of training pairs seems to make it harder and not easier to learn the linear relationship between shape and mapping. This is first evidence that the 5 shape-mapping contingencies are learned independently (as predicted by the Mixture-of-Kalman-Filters) and it is not the linear relationship that is learned.

This conclusion can be further supported by analysing the transfer of learning. Here we see that both the 5-Pair and the 2-Pair conditions appear to have very similar effects. If participants would have been able to extract the linear rule underlying the training conditions, we predicted that extrapolation of learning in the 5-Pair Condition would be more linear since the participants had more evidence for the linear nature of the correlation between shape and mapping in this case. However, when comparing the 2-Pair and the 5-Pair conditions for the extrapolation shapes, the results seem very similar. That is, for circular shapes (*ρ* close to 1.0) the applied mapping stays more or less constant compared to the nearest training pair. For more spiky shapes than those trained, at first glance it seems that in the 5-Pair Condition the extrapolation might be more linear. However, the hypothesis that the behavioural correlations between mapping and shape would be linear was rejected for all participants except one in the 5-Pair Condition and two in the 2-Pair Condition. These tests were again performed by testing for differences in the regression residuals using an ANOVA for each individual participant and correcting for multiple comparisons (Bonferroni). This means that for both the 2-Pair and the 5-Pair Condition there is further evidence that transfer of learning does not necessarily occur in a linear fashion.

Instead the Mixture-of-Kalman-Filters performs quite well in predicting the generalisation patterns. Though again it slightly overestimates the amount of learning, which leads to significant differences in the residuals of the model for 3 of the 6 subjects in the 2-Pair Condition and all the participants in the 5-Pair Condition. However, again the comparisons of *R* ^2^ for the two models favoured the Mixture-of-Kalman-Filters at least for the 2-Pair Condition (2-Pair Condition signed-rank p = 0.03; mean $R_{\text{MKF}}^{2}$ = 0.85; mean $R_{\text{Lin}}^{2}$ = 0.44). For the 5-Pair Condition a better performance of the Mixture-of-Kalman-Filters Model could not be confirmed (5-Pair Condition signed-rank p = 0.13; mean $R_{\text{MKF}}^{2}$ = 0.65; mean $R_{\text{Lin}}^{2}$ = 0.27).

### Experiment 3

The previous experiments provide little evidence for a linear extrapolation beyond the trained shape-mapping pairs. If anything, it appears to occur for shapes that are spikier compared to the trained shapes. To further explore whether there might be some linear extrapolation on this side of the shape-scale, we performed one more experiment for which the training was conducted with three shape-mapping contingencies and shapes that were overall more circular (*ρ* > 0.375). That is, the centre of the training regime was shifted towards more circular shapes, providing room for extrapolation of the learned relationship when spikier shapes were next presented. Furthermore, the slope between the required mapping and the shape parameter was increased (see Fig. 4C) to better distinguish between linear extrapolation and nearest-training-pair extrapolation. This slope was 1.5 times as large as in the previous experiments. The exact mapping pairs that were used for training in Experiment 3 were: (*ρ* = 0.375, mapping = −37.5mm), (*ρ* = 0.625, mapping = 37.5mm) and (*ρ* = 0.875, mapping = 112.5mm). As in Experiment 2, participants were informed about the fact that the shapes were important for the task such that they would have to adjust their behaviour depending on the shape. However, participants were not informed about the specific shape-mapping contingencies.

The results for Experiment 3 are shown in Fig. 4C. For the three shape-mapping pairs learning occurs again to a relatively large extent (65%). As noted above, Experiment 3 was designed to particularly test for linear extrapolation when using more spiky shapes. Fig. 4C clearly shows that for more spiky shapes the behavioural mapping does not follow the linear extrapolation prediction. This was confirmed by the ANOVA on the regression residuals for each participant. Instead, again the transfer of learning to extrapolation shapes seems to occur in a way that the mapping for the more extreme shapes is the same as for the nearest trained shape (see also Experiment 2).

The Mixture-of-Kalman-Filters Model performed much better at predicting the generalisation results (signed-rank p = 0.002; mean $R_{\text{MKF}}^{2}$ = 0.85; mean $R_{\text{Lin}}^{2}$ = 0.57). Though, again it has to be noted that for all participants there were significant differences in the residuals, indicating that the fits were also not perfect even for the Mixture-of-Kalman-Filters. As before, this is mostly due to a misestimation of the model for the learning extent. In contrast however, the Linear Generalisation Model does not make any predictions about the amount of learning, but instead treats it as a fitted/free parameter.

### Learning rates

The results of Experiment 2 and 3 both indicate that at least extrapolation does not occur in a linear fashion. A possible explanation is that participants learned each training pair separately, instead of learning the underlying structure of the conditions. That the amount of learning for the 5-Pair Condition in Experiment 2 was less than for the 2-Pair Conditions is in line with this explanation. Learning the training pairs raises the possibility that the learning for one pair interferes with the learning of a neighbouring pair through local generalisation. Thus with more pairs in the training set there could also be more interference in the learning. To investigate this more closely, it is useful to look at the learning rates as a function of the number of training pairs.

To more easily compare the learning rate across the different training conditions, we calculated the normalised learning extent versus the trial number in the first training block for each condition in Experiment 2 and 3. The normalised learning extent is expressed by the ratio between the behavioural mapping which is currently applied by the participant and the one that is required (depending on the particular target shape for each trial). Note, that learning often was not immediate since it required the participant to be aware of the role of the different shapes. For a fair comparison across conditions we therefore determined for each participant the point at which learning actually started. This was done by moving a sliding window (width 50 trials) across the normalised training extent and performing a t-test for each point in time. The trial at which the t-test indicated that the normalised learning extent after that trial was significantly above zero (at an *α*-level of 0.000625 for Bonferroni correction) was taken as the time point at which participants realised the role of the shape and started to learn the mappings. Next, the learning curves for each participant were fit with an exponential function (1 − *e* ^−*λt*^) with *λ*, the learning rate, as the only free parameter and *t* representing the trial number, before averaging the fitted curves across participants.

**Fig 5:**
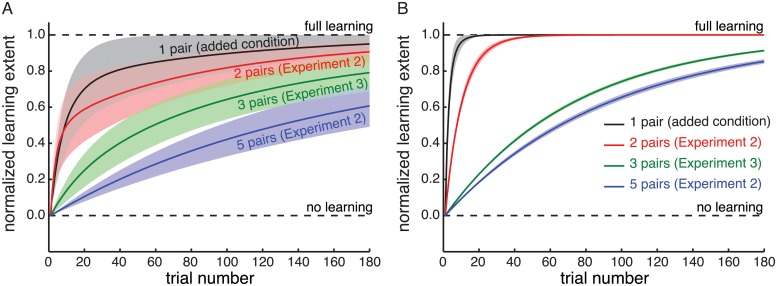
Learning rate results. The learning trend versus trial number for the conditions of Experiment 2 and Experiment 3 plus an added condition in which only one shape-mapping pair was used in the training. A) Experimental Results. B) Predictions for the Mixture-of-Kalman-Filters Model. Shaded areas represent the standard error across participants. The learning rate drastically decreases with increased numbers of training pairs and the model qualitatively captures this finding.

The results of this analysis are depicted in Fig. 5A. Next to the conditions from Experiment 2 (2-Pair—red; 5-Pair—blue) and Experiment 3 (3-Pair—green) a baseline condition with only one shape-mapping pair (*ρ* = 0.5; mapping = 50mm; 4 participants) was added for comparison. The training and test paradigm for the baseline condition was otherwise the same as for the conditions of Experiment 2 and 3. The extent of learning versus the trial number for this baseline condition is indicated in black. Solid lines indicate the mean across participants and shaded areas indicate the standard errors. Note, that only results for participants that actually learned the mappings are taken into account for this analysis. Nevertheless, it is clearly visible from Fig. 5 that the learning rate decreases systematically with the number of mappings that the participants have to learn simultaneously. This indicates interference between the different training pairs and it suggests that learning these mappings is not structural but each separate pair is learned separately.

To compare these results to the Mixture-of-Kalman-Filters Model, we re-ran the model simulations for each participant using the trial-sequence from the point at which each participant started to learn. More importantly, to verify whether the slow-down of learning with an increased number of training pairs is an emergent property of the model, the simulations for all participants and experimental conditions were done using the same ratio between *σ*_*y*0_ and *σ*_*process*_ in this case (see Bayesian Learning Model in Materials and Methods). In other words, we fixed the only free parameter of the model for this analysis. This was done since the previously fitted values may directly incorporate different learning rates for the different conditions. In that case the different learning rates would be a property of the fits rather than an emergent property of the model itself. For the fixed ratio between *σ*_*y*0_ and *σ*_*process*_, we chose the median fitted ratio across all participants and all experimental conditions. For these simulations we again fitted exponential functions to the normalised learning extent to derive the overall learning rate across training pairs. Note, that this analysis cannot be performed for the linear generalisation hypothesis because this hypothesis does not make any quantitative predictions for the learning rate.

The results of this analysis using the Mixture-of-Kalman-Filters Model are shown in Fig. 5B. The model clearly captures the slow down of learning with increased number of training pairs at least qualitatively. In the model the interference comes from the generalisation for one particular training shape affecting behaviour for neighbouring shapes (see Equation 5). In other words the interference between shapes with the increased number of training pairs is indeed an emergent property of the model.

## Discussion

We investigated the learning of the association between previously irrelevant target shape cues and behaviourally relevant visuomotor mappings. The novelty of the current study is that both the shape cue and the visuomotor mappings varied in a continuous manner and the training was focussed on learning a functional relationship between the shape and the corresponding mapping—here a linear relationship. To test if participants learned this underlying linear relationship, we looked for generalisation of learning to novel shape-mapping pairs. The results first show that learning the shape-mapping association occurs. Secondly, the results indicate that interpolation between two trained shape-mapping pairs occurs in a more or less linear manner (Experiment 1). However, extrapolation of learning, i.e. for shapes beyond the range of shapes that were explicitly trained, did clearly not occur in a linear fashion indicating that participants did not learn the linear relationship between mapping and shape. Rather, the results are quite consistent with the Mixture-of-Kalman-Filters Model. This model is based on previous findings in the sensorimotor behaviour literature (e.g. the Kalman filter and Mixture-of-Experts approaches; [24–27]). Generalisation for the Mixture-of-Kalman-Filters Model occurs by learning for the separate training shapes influencing the mapping for neighbouring shapes. Importantly, the Mixture-of-Kalman-Filters Model is the first model that links the discrimination threshold between shapes to the generalisation pattern, instead of treating the generalisation gradient as a free parameter (see e.g. [28–30]).

The extrapolation pattern predicted by the model is one in which participants apply more or less the mapping that is associated with the trained shape which is closest to the currently presented shape as observed in the results. Furthermore, the Mixture-of-Kalman-Filters predicts slower learning rates with an increased number of training pairs as was also observed experimentally. This is because the learning processes for the individual training shapes interfere more and more with an increased number of training pairs, due to learning for one shape also affecting learning for other shapes along the same scale (Equation 5). This interference will increase with an increased overlap of generalisation from the training shapes. In our experiments an increase of the number of conditions that needed to be learned led to a decrease in inter-training-pair spacing and thus more overlap. This fact that learning times systematically increase with a decrease in spacing between the training conditions, even if they represent a simple relationship, can have major implications for daily life conditions. For instance, when acquiring bifocal glasses the learning time to get used to them can take quite a bit longer than for glasses having only a single focal length. Consistent with our model this increased familiarisation time would be due to the large discontinuity between the two focal areas leading to a large amount of learning overlap. This is a possible reason why some people tend to switch back to having two separate pairs of glasses for reading and normal daily activities.

For learning to occur, participants needed to be aware of the role that the shape-cue was playing. At least for Experiment 1, where participants were not informed about the role of the target shape prior to the experiment, only those participants learned who consciously noticed the role of the shape. This indicates that learning the shape-mapping relationship is not an entirely automatic process. To gain more insights into the extent to which the learning involved explicit knowledge, we conducted a control experiment in which we investigated the amount of transfer between the trained and the untrained hand (for details see Supporting S3 Text). For adaptation to a simple shift in the visual feedback, transfer has been shown to exist but only to a very limited extent. For an additional explicit component, it should however not matter if the task is performed with the trained or with the untrained hand, since the explicit knowledge can easily be applied to both. We conducted two conditions, one with two training pairs and one with only one (thus mimicking standard visuomotor adaptation). Compared to the one-pair condition, the results for the two-pair condition showed a substantial amount of transfer between the hands, confirming the role of explicit learning of the contextual cues. The strong influence of explicit learning can account for many of the contextual cue effects reported in the literature and may account for the fact that we never fully implicitly compensate for our own glasses when putting them on [31].

Note that the ease of learning contextual cues in the current and previous experiments, seems in contrast to studies in which contextual cues were informative about force fields or visuomotor feedback rotations during reaching. In many of the latter studies, contextual cues were only effective under very specific circumstances (see e.g. [32–39]) whereas for the simpler amplitude gains or shifts of the visual feedback as used here, contextual cues are generally highly effective [8–18]. One of the major differences between the two types of studies is that the error information in force-field or visuomotor feedback rotations conditions is highly dynamic. That is, the sense of error increases during single movements, emphasising the role of online visual feedback to correct the movements in order to reach the target. Contextual cues would have to compete with the continuous feedback and dynamic error processing both in terms of attention—focussing on the feedback potentially makes it harder to apply an explicit strategy [40]—as well as the effectiveness for solving the task in general (to reach the target, correcting for errors online is in principle sufficient). In those more complex environments, learning the contextual cues could also suffer interference from variability in movement speed, which would add noise to the perceived effect-size of the perturbations. In any case, a recent study suggests that some amount of explicit learning by adjusting an aim-point, a learning strategy that could account for the results reported here, is likely the case in any visuomotor adaptation task [40]. Future research will have to point out whether the mechanism behind learning contextual cues in more dynamic feedback environments potentially follows a similar mechanism as the one suggested here.

In light of the major role of explicit awareness for learning the contextual cues in the current study it is surprising that the generalisation results are so consistent across participants. This suggests that there is a default strategy for the interpolation and extrapolation to shapes that were not explicitly used in the training without participants particularly “choosing” a strategy for the new generalisation shapes. Otherwise we would have expected the generalisation results to be much more variable across participants. Interestingly, even participants who described their extrapolation strategy as a continuous scaling from shape to response in the debriefing questionnaire (i.e. pointing more extremely left or right compared to training for more extreme shapes) often produced a categorisation-like generalisation behaviour. Thus, it is very likely that the generalisation pattern is not fully governed by simply applying an explicit rule. Moreover, studies of cognitive function learning in which a stimulus-response mapping is learned from a limited number of examples, much like in the current study but without requiring a visuomotor response, have shown linear extrapolation from the training conditions (see e.g. [29, 41]). The difference in generalisation between function learning studies and our results suggests that at least the process for generalising contextual cues in a visuomotor task is not the same as could be expected if a fully cognitive stimulus-response mapping was learned. In any case, the Mixture-of-Kalman-Filters seems to capture the learning process for contextual cues in visuomotor tasks and the “default generalisation strategy” quite well. That is, separate learning processes are maintained depending on the target shapes with some interaction between the learning processes. Note, that the Kalman-filter is often regarded as a model for automatic/implicit learning. The good correspondence between the Mixture-of-Kalman-Filters and our results thus suggests that, though explicit knowledge about the role of the contextual cues is necessary to separate the cue conditions, the learning process associated with each cue has an implicit learning component that is captured by the Kalman-filter. Note, however, that the model predictions were also not always perfect, sometimes leading to significant residuals between model and results for individual participants. This may point to influences other than the automatic update within each Kalman Filter.

Why some participants learn and others do not and why some participants learn sooner than others, is one aspect that the model cannot explain. That is, the model only describes a potential mechanism of how learning occurs once the participants have already realised the importance of the shape cue, not how they come to realise it. To describe when participants start using a particular cue, some categorisation and function learning models include dimensional attention parameters that could make the shape switch from being an irrelevant to a relevant cue, depending on for instance the context (see e.g. [42–44]). In principle, such an attention parameter could easily also be added to the Mixture-of-Kalman-Filters Model to describe the switch when participants start using the shape-cue. However, such an attention parameter would be another free parameter used for fitting the data and as such has no explanatory power.

To summarize, when confronted with a new continuous correlation between a previously irrelevant shape cue and the necessary visuomotor mapping in order to hit the target, learning of this correlation does not occur automatically but rather awareness of the correlation seems necessary to learn. Furthermore, participants did not try to extract a general rule from the training conditions. Rather, it seems that the participants learn the set of separate training pairs and, in order to generalise to new shapes, mix the known conditions according to their similarity with the current shape. This principle is captured quite well by a Mixture-of-Kalman-Filters for which each shape along the continuous cue-scale is coupled with its own learning process.

## Materials and Methods

### Ethics statement

The experiments were approved by the ethics committee of the department of medicine of the University of Tübingen (Germany). All participants gave informed consent.

### Apparatus

Stimuli were displayed on a large back-projection screen (220 by 176 cm) in an otherwise dark room. Participants were seated behind a custom-made rack (see Fig. 2A). On the first level of the rack a graphics tablet (WACOM Intuos 3 A3-wide; active area 48.8 by 30.5 cm and a grip pen) was placed in order to record the pointing behaviour of the participants. A second level of the rack draped in black cloth prevented the participants from seeing their own arm or the graphics tablet while performing the pointing task. The head movements of the participants were restricted by a chin rest at a viewing distance of 53.5 cm from the screen. The visual stimuli were implemented in C using OpenGL. Scales on the screen were mapped one-to-one to scales on the tablet such that only the centre portion of the screen was used to display target shapes. For a complete mathematical description of the target shapes see the Supporting S1 Text and Fig. 1.

### Stimuli and task

Participants used their preferred hand for pointing. A trial was initiated by tapping with the pen within a horizontally centred semi-circular area (radius of 25 mm) on the lower edge of the tablet’s active area (see Fig. 2). This area was also indicated haptically by a physical ring attached to the tablet. Participants were told to hold their non-preferred hand on or near this ring to be able to find this position more easily with their preferred hand used for pointing without vision. On the screen a white horizontal line provided a visual reference for the vertical position of the starting zone.

After trial initiation a target shape was displayed and the horizontal white line disappeared. The inner and outer radii for each target (see Fig. 1) were chosen such that the surface area matched that of a circular disk with radius *R* = 1.3 deg (12.5 mm). The target location was drawn randomly from within a square region of 10.7 by 10.7 deg centred 11.6 deg above the starting zone. Participants’ task was to try and tap in the corresponding location on the graphics tablet as accurately and as quickly as possible within a 1 second time limit. After each trial participants received feedback about the location where they pointed in the form of a high contrast Gaussian blob on the screen. The standard deviation of the Gaussian blob was only 3.5 mm (0.37 deg) on the screen in order to provide visually very reliable feedback. By manipulating the location of this feedback relative to the pointing position on the tablet we can simulate different visuomotor mappings. That is, different motor responses are required to bring the feedback on the target location. For simplicity, we chose to manipulate the feedback in only the horizontal direction. Additional feedback was given in form of credit points, the amount of which depended on the absolute visual error between target location and feedback location. Participants scored 100 points if the absolute pointing error was below 1 cm (1.07 deg visual angle), 50 points when the error was between 1 and 2 cm, 25 points between 2 and 3 cm error and 0 points otherwise. The scoring region would be indicated on the screen by showing a bullseye pattern across the target along with the feedback (Fig. 2). The cumulative score was displayed on the screen at all times.

In order to ensure that movements become more and more automatic with practice a time limit of 1 sec from trial initiation was set for participants to complete a single reaching movement. If the participant did not finalise the pointing movement within this limit, the trial terminated with a message “too slow” and 500 points were subtracted from the total score.

### Training and catch trial procedure

Before starting with the experiment participants were familiarised with the task and setup. They were allowed a few practice trials for which the feedback was veridical and the target shape was a triangle. That is, it was a shape different from the shapes used for training and testing in the main experiments.

After the practice trials, participants performed 5 experimental blocks each consisting of 180 trials. For Experiment 1, these learning trials could embrace one of two possible training shape-mapping combinations: one training combination was a shape with *ρ* = 0.125 (i.e. a relatively spiky shape) and a mapping of −75 mm (i.e. visual feedback was shifted by 75 mm to the right with respect to the actual pointing location, such that the participant would have to point 75 mm to the left of the target in order to bring the feedback on target as in Fig. 2C); the other training combination was shape *ρ* = 0.875 (i.e. an almost circular shape) and a mapping of +75 mm (note that we counterbalanced the sign of mappings paired with the two different shapes across participants: the results for participants with the reverse pairing were mirrored accordingly before averaging across participants). These two different trial types were presented in random order in each experimental block.

The fifth and last block differed from the other four. In the fifth block participants performed 240 trials of which 180 were training trials as before. The other 60 trials were catch trials in which we tested the transfer of training to other target shapes. On catch trials participants did not receive any visual feedback indicating the pointing end-point and instead of a score a question mark appeared. The points scored during these catch trials, based on vertical error alone instead of absolute error, were given as a bonus at the end of the block (i.e. at the end of the experiment). In this way the catch trials did not provide any useful information for learning. If, however, the participant exceeded the time limit of 1 sec for a catch trial the penalty of 500 points was incurred immediately. The target shape *ρ* on these catch trials was either: 0.01, 0.25, 0.375, 0.625, 0.75 or 1.0 and for each of these values there were 10 catch trials. The trial-order in the fifth block was also randomized except that care was taking that catch trials did not occur within the first 30 trials of this block.

Participants were required to take a 5-minute break after each experimental block in order to prevent fatigue from influencing the data. During these breaks participants had access to their scores from previous blocks as well as to a hi-score list in which the 10 best scores over all participants were listed anonymously. In this way, participants could track their own increase in performance over consecutive blocks and were motivated to perform better on the next block.

In Experiment 1 twelve observers participated who had normal or corrected-to-normal vision and no known history of visuomotor deficits. The participants were not informed about the different shapes nor about the shape’s meaning in terms of required pointing behaviour. After the experiment the participants filled in a questionnaire asking them whether they had noticed the different shapes and their contingencies with the required pointing behaviour. Eight out of the twelve participants indicated they had realized the meaning of the different shapes.

### Experiments 2 and 3

In Experiment 2, we tested two different training conditions, each for a new group of 8 participants who had not participated in Experiment 1 nor in the other condition of Experiment 2. In the 2-Pair Condition, two training shape-mapping contingencies were used (*ρ* = 0.25, mapping = −50 mm and *ρ* = 0.75, mapping = +50 mm). In the 5-Pair Condition, five shape-mapping pairs spanned the same training range as the 2-Pair Condition: (*ρ* = 0.25, mapping = −50mm), (*ρ* = 0.375, mapping = −25mm), (*ρ* = 0.50, mapping = 0mm), (*ρ* = 0.625, mapping = 25mm) and (*ρ* = 0.75, mapping = 50mm).

In Experiment 3, six participants were trained on three shape-mapping pairs: (*ρ* = 0.375, mapping = −37.5mm), (*ρ* = 0.625, mapping = 37.5mm) and (*ρ* = 0.875, mapping = 112.5mm).

In Experiment 2 and 3 participants were informed about the fact that different shapes would need different pointing behaviours. However, participants were not informed about the particular shape-mapping contingencies, which they had to deduce themselves.

### Bayesian learning model

We used the approach of Bayes-optimal learning in which new sensory information (e.g. through sensory feedback) is combined with prior experiences (e.g. from previous trials) in a statistically optimal way in order to obtain the most precise possible response (the posterior estimate). In the Bayesian framework such optimal combination occurs if the sensory information and the prior are each weighed according to their respective precisions. In Bayes-optimal learning this combination of sensory input and prior knowledge becomes an iterative process: the posterior at one time-point serving as a basis for the prior for the next time-point, etc. The Kalman Filter [45] is such an adaptive procedure that takes into account the precision of the current internal estimate of the required mapping (the “prior” as it were, at that specific point in time) as well as the precision of the incoming information in the form of sensory feedback, which is used to update that estimate. Here we modelled the learning of the shape-mapping contingencies as a Mixture-of-Kalman-Filters in which each shape is coupled to its own Kalman Filter.

**Fig 6:**
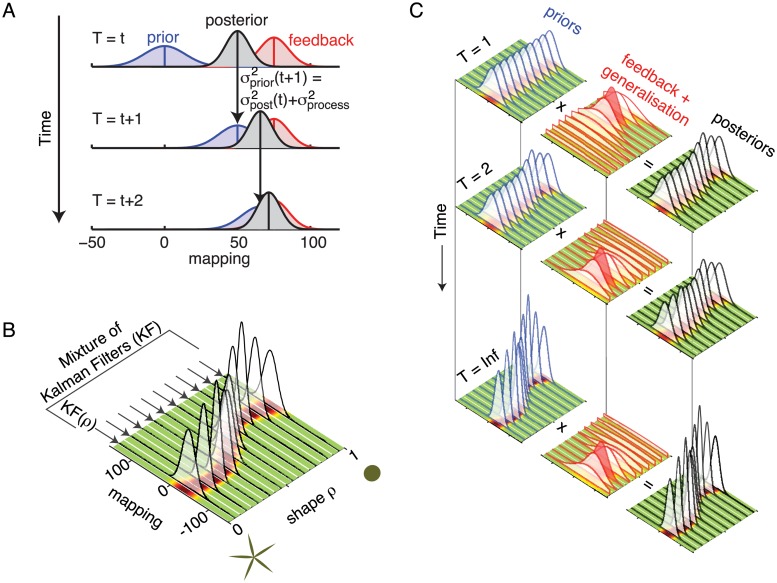
The Mixture-of-Kalman-Filters Model. A) The updating process of a single Kalman filter. On each trial a prior internal estimate (blue) is combined with sensory feedback (red) to obtain a posterior estimate (black) of the mapping that should have been used. This posterior estimate serves as the prior estimate for the next trial through propagation in time, which is not noise free (thus adding *σ*_*process*_). B) Mixture-of-Kalman-Filters. For each shape along the shape scale a separate Kalman Filter updates the associated mapping. C) Mixture-of-Kalman-Filters updates over time. The initial prior estimates for each Kalman Filter (left) are being updated on each trial according to the shape seen and the feedback received (middle) to obtain the posterior estimates (right). The feedback is most precise for the shape on the current trial (indicated in red) and the precision of the feedback decreases for shapes further away along the shape scale. After only a few iterations the pattern for generalisation already starts to emerge in the estimates of the Mixture-of-Kalman-Filters Model.

Assuming that the noise in the estimates is Gaussian, the mathematical description for the Kalman filter for each single shape (Fig. 6A) is as follows:

$$\begin{array}{c} \text{Kalman}\;\text{gain:}\;K\left(t\right)=\frac{\sigma_{prior}^{2}\left(t\right)}{\sigma_{prior}^{2}\left(t\right)+\sigma_{Y}^{2}} \end{array}$$ (1)

$$\begin{array}{c} \text{Posterior}\;\text{estimate:}\;X_{post}\left(t\right)=X_{prior}\left(t\right)+K\left(t\right)\left(Y\left(t\right)-X_{prior}\left(t\right)\right) \end{array}$$ (2)

$$\begin{array}{c} \text{Posterior}\;\text{uncertainty:}\;\sigma_{post}^{2}\left(t\right)=K\left(t\right)\sigma_{Y}^{2} \end{array}$$ (3)

$$\begin{array}{c} \text{Progression}\;\text{to}\;\text{T}\;\text{=}\;\text{t+1:}\;\sigma_{prior}^{2}\left(t+1\right)=\sigma_{post}^{2}\left(t\right)+\sigma_{process}^{2} \end{array}$$ (4)

Here *X*_*prior*_(*t*) and *X*_*post*_(*t*) represent the prior and posterior estimates of the required mapping, respectively. That is, *X*_*post*_(*t*) is the estimate obtained by combining the information from the previous estimate, the prior *X*_*prior*_(*t*), and the feedback after the movement has been completed, *Y*(*t*), providing additional information about what the mapping should have been (see also Fig. 6A). $\sigma_{prior}^{2}(t)$, $\sigma_{Y}^{2}$ and $\sigma_{post}^{2}(t)$ represent the respective uncertainties of the prior, the feedback, and the resulting posterior estimates. *K*(*t*) denotes the Kalman gain with which the estimates are updated with respect to the feedback.

$\sigma_{process}^{2}$ is the process noise involved in propagating the current estimate (the posterior) into a future estimate (the prior for the next iteration). The propagation is based on an assumption how the system should develop over time (in this case we used the assumption of zero change) and is not noise free. This step furthermore ensures that without further feedback, the internal estimate of what the current mapping should be, will grow more and more uncertain over time (leading to an increased Kalman gain; Equation 1). In other words, the process noise is an important factor in determining the learning rate involved in tasks such as the one described here.

To describe the behaviour of the Mixture-of-Kalman-Filters across the target shapes, it is important to note that the perception of the shape is noisy and thus cannot singularly influence one filter only. That is, learning for one shape will also affect the filters of neighbouring shapes in a graded fashion. We modelled this by increasing the uncertainty of the feedback for any particular Kalman Filter as a function of the difference in shape-space between the corresponding shape for that filter *ρ* and the current shape with which the system is being presented *ρ*_*t*_ along the continuous axis (Fig. 6C).

$$\begin{array}{c} \text{Generalisation:}\;\sigma_{Y}^{2}\left(\rho \right)=\sigma_{y0}^{2}\;\left(1+\frac{{\left(\rho -\rho_{t}\right)}^{2}}{2\sigma_{\rho }^{2}}\right) \end{array}$$ (5)

Here *σ*_*y*0_ is a constant based on the visual reliability of the feedback and the motor noise and *σ*_*ρ*_ represents the noise in shape perception. Note that we chose a similar dependence on shape-difference for the generalisation function, which is normally used in Gaussian distributions ($\frac{{\left(\rho -\rho_{t}\right)}^{2}}{2\sigma_{\rho }^{2}}$), in order to directly link the generalisation across shapes to the shape discrimination threshold *σ*_*ρ*_ (see Supporting S2 Text). The quadratic dependence on shape difference (*ρ*−*ρ*_*t*_) furthermore ensures that there are no discontinuities in the pattern of generalisation.

The response when being presented with a certain target shape on any individual trial, should of course take into account the learned associations between target shape and mapping. That is, on each trial a certain target shape is presented and a response has to be chosen with the correct mapping that brings the hand to the target. The correct response will depend on the state of the Kalman Filter corresponding to the target shape that is currently being presented. However, as noted above perception of the shape will not be noise free. This means that also the uncertainty of the current shape (*σ*_*ρ*_) needs to be considered. Mathematically, this can be modelled as a weighted average, weighing the response of each individual Kalman Filter KF(*ρ*) according to the likelihood that its associated target shape is the one currently being presented:

$$\begin{array}{c} \text{Shape}\;\text{likelihood:}\;P\left(\rho |\rho_{t}\right)=N\left(\rho |\rho_{t},\sigma_{\rho }\right) \end{array}$$ (6)

$$\begin{array}{c} \text{Model}\;\text{response:}\;\text{MR}\left(\rho_{t}\right)=\sum_{\text{KF}(\rho )}E\left(\text{KF}\left(\rho \right)\right)P\left(\rho |\rho_{t}\right) \end{array}$$ (7)

In Equation 7, *E*(KF(*ρ*)) represents the expected response from each Kalman Filter in the mixture, i.e. its prior estimate. Note that the response selection and the updating process of the Kalman Filter can be considered as two independent steps in the model.

We used the model to make predictions for the generalisation patterns as well as the learning rates and how they depend on the number of training pairs. For the generalisation patterns, the predictions were derived by fitting the model to the sequence of trials, before testing its response on the generalisation conditions. In this case the model had one free parameter per participant, i.e. the ratio between *σ*_*y*0_ and $\sigma_{process}^{2}$ which determines the learning rate. *σ*_*ρ*_ instead was obtained from a separate experiment in which we determined the discrimination thresholds for the different shapes (see Supporting S2 Text). Predictions for the influence of the number of training pairs on the learning rates were made using a fixed ratio between *σ*_*y*0_ and $\sigma_{process}^{2}$ for the model simulations.

### Model comparison

For the linear generalisation prediction a linear regression was performed on the training results of the last block of trials for each participant. This means that for each participant there were two free parameters for the linear generalisation hypothesis (offset and slope). To test the hypothesis for linear generalisation directly, ANOVA’s were performed on the residuals of all trials in the last trial-block, i.e. including catch trials. If the learning and transfer of learning occurs in a linear fashion there should not be any difference in the residuals between the different shapes used in the experiment. Thus, if the ANOVA leads to a significant result the hypothesis for linear generalisation can be rejected.

For the Mixture-of-Kalman-Filters Model, we simulated the sequence of responses using the trial sequences (i.e. the order of training shapes) exactly as they had been used for the individual participants in the experiment (including catch trials). That is, simulations were made for each participant individually. The best fit for each participant was obtained by fitting the ratio between *σ*_*y*0_ and *σ*_*process*_ that determines the learning rate (one free parameter per participant). The fitting procedure was done by minimising the sum of mean squared errors (between human and model responses) across trials using a grid search to find the global minimum. For finding the best fit, only trials in the last block were used to keep the fitting procedure as similar as possible to the procedure used for the linear generalisation hypothesis. However, as mentioned above, model simulations themselves were performed using the trial sequence across all blocks for each individual participant. For the simulations 100 Kalman Filters were included in the mixture, spanning the available shape-space in its entirety (from *ρ* = 0.01, a very spiky shape, to *ρ* = 1.0 for circular shapes).

*R* ^2^ were computed for both models for each participant individually and were compared across participants using a signed-rank test.

## Supporting Information

S1 Text Mathematical description of the target shapes. (PDF)

S2 Text Verifying perceptual linearity of the *ρ* shape-scale / measuring *σ*_*ρ*_. (PDF)

S3 Text Intermanual transfer of learning. (PDF)
