# Supplementary material for: Mapping Shape to Visuomotor Mapping: Learning and Generalisation of Sensorimotor Behaviour Based on Contextual Information
Source: PLoS Comput Biol. 2015 Mar 27;11(3):e1004172. doi: 10.1371/journal.pcbi.1004172 (PMC4376781; doi:10.1371/journal.pcbi.1004172)
Supplement: S1 Text — (PDF) [file pcbi.1004172.s001.pdf]

S1 Mathematical description of the target shapes

The target shapes that were used in the experiments ranged from circular to spiky shapes (see Figure 3) and can be described mathematically (i.e. no mesh morphing was used). The separate shapes differ in the extent to which the spikes reach (the outer radius r_o) and in the extent to which the indents reach (the inner radius r_i; see Figure 1). By taking the ratio between these inner and outer radii, every shape along this "spikiness" scale can be characterised by a single shape parameter ρ:

$$\text{shape parameter:} \quad \rho = \frac{r_i}{r_o}$$

A shape parameter of 1 means that the inner and outer radii are equal and therefore refers to a normal disk. Very small ρ means that the outer radius is much bigger than the inner radius and the shape is very spiky.

The spikes of the shapes were made slightly bulgy in order to prevent the shape from hardly having any surface for small ρ. The bulgy spikes were obtained by drawing exponential curves in the polar coordinate system between the points on the inner and outer radii. This means that the distance from the centre of the shape r varies as a function of the angle θ between two neighbouring points on the respective inner and outer radii. This can be mathematically described as:

$$r(\theta) = r_i \; e^{\left(\frac{\theta - \theta_i}{\theta_o - \theta_i}\right) \ln\left(\frac{r_i}{r_o}\right)} \qquad \text{for} \quad \theta \in [\theta_i, \theta_o]$$

Here θ_i is a single point on the inner radius, i.e. exactly at an indent of the shape, and θ_o a neighbouring point on the outer radius, i.e. exactly at the peak of the neighbouring spike. θ_i and θ_o therefore depend on the number of spikes in the shape. We used shapes with 5 spikes in the experiments, which means that θ_i and θ_o differ by $360/(2 \times 5) = 36$ deg rotation angle.

From these formulas it is clear that for a constant ρ there are multiple possible combinations of r_i and r_o and these combinations differ only in the overall scale of the shape. Real changes in shape are only possible by changing ρ but in order to do so either r_i, r_o or both should change. Fixing either r_i or r_o leads to problems in experimental design when ρ is small. If r_i is fixed r_o has to be large in order to obtain a small ρ which means that we get a very large shape. If instead r_o is fixed r_i has to become very small for small ρ and the spikes will become very thin, which might mean that the shape will also

decrease in visibility since the overall surface area becomes smaller. Therefore, rather than fixing either r_i or r_o, we chose to control for a constant surface area across the shapes-scale. To control for the surface area the r_i and r_o where set as follows:

$$r_o = R\sqrt{\frac{2\ \ln(\rho)}{\rho^2 - 1}} \qquad \text{for} \quad 0 < \rho < 1$$

$$r_o = R \qquad \text{for} \quad \rho = 1$$

$$r_i = \rho\ r_o$$

Where R represent the radius when r_i and r_o are equal, i.e. when $\rho = 1$ and the shape is a normal disk. In the experiment R was set to 1.3 deg (12.5 mm). Note that $\rho = 0$ represents a singularity for which the spikes of the shape become infinitely thin and long and the equations above do not provide an adequate solution. Therefore in the experiments the smallest ρ used was set to 0.01.

We verified empirically that the shape-space defined in this way was approximately perceptually linear (see Supporting Text S2 for details). In the main experiment, we chose for each shape from this linearly perceived shape space a corresponding mapping that varied linearly with the shape.
