# Supplementary material for: Mapping Shape to Visuomotor Mapping: Learning and Generalisation of Sensorimotor Behaviour Based on Contextual Information
Source: PLoS Comput Biol. 2015 Mar 27;11(3):e1004172. doi: 10.1371/journal.pcbi.1004172 (PMC4376781; doi:10.1371/journal.pcbi.1004172)
Supplement: S2 Text — (PDF) [file pcbi.1004172.s002.pdf]

S2 Verifying perceptual linearity of the ρ shape-scale / measuring σ_ρ

In order to train participants on a linear relationship between target shape and visuomotor mapping, the continuous shape scale needs to be perceptually linear. We verified that this was indeed the case in three control experiments. First, perceptual linearity of the shape-scale means that the different shapes along the scale need to be equally distinguishable from their respective neighbours. That is, the shape discrimination threshold should be constant along the shape scale. To verify if this was indeed the case for the shape scale used in the main experiments, we measured and compared Just Noticeable Differences (JND) for several different standard shapes along the scale (Control Experiment 1). Note that the obtained JNDs in terms of the shape parameter ρ additionally provided an empirical value for the noise in shape perception (σ_ρ) for use in the Bayesian learning model as described in the main text.

Second, perceptual linearity of the shape-scale means that the perceived scaling of the shapes should be linear in terms of ρ. This was verified in two further control experiments. In the Control Experiment 2 dissimilarity ratings were collected for each possible pair of the 9 shapes used in the main experiment. These dissimilarity ratings were used in a non-metric Multi-Dimensional Scaling (MDS) analysis to obtain the perceived spacing between the shapes. In Control Experiment 3 the perceptual scale for the shapes was measured more directly, by having participants place individual shapes on the scale between the two most extreme shapes ($\rho = 0.01$ and $\rho = 1.0$).

The results for these three control experiment were very consistent and indicated perceptual linearity for the shape-scale in terms of ρ. The three experiments are described in more detail below.

Control Experiment 1: Measuring σ_ρ

We measured the JNDs in terms of ρ for three different standards: $\rho = 0.1$, $\rho = 0.5$ and $\rho = 0.9$ using the method of constant stimuli in a 2-Alternative-Forced-Choice (2-AFC) task. Participants were presented two target shapes in two separate intervals and their task was to indicate which of the two intervals contained the spikier shape (see Figure S1B, top). One stimulus interval showed one of the standards and the other interval showed one of the comparison stimuli in counterbalanced order. The comparison stimuli were one of ± 0.15, ± 0.1, ± 0.05, ± 0.025 or 0.0 relative to the standard, provided the

ρ for the comparison shape still fitted within the range $[0.01, 1.0]$.[1] The shapes were shown for 1 sec each and after each stimulus interval a mask consisting of 105 small coloured rectangles was shown for 1 sec (Figure S1A). The rectangles within the mask were randomly sized (the lengths of the sides were randomly chosen between 0.0 to 1.2 deg using a uniform distribution) and randomly positioned within a 6.4×6.4 deg square.

Each standard-comparison pair was tested 10 times and trials were presented in random order. Psychometric curves (cumulative Gaussians) were fitted to the proportions of comparison shape perceived as less spiky for each standard shape. JNDs were obtained by taking half the distance between the 25% and the 75% percent cutoff points of the fitted psychometric curves. Assuming a Gaussian Distribution, the JND obtained in this fashion relates to σ_ρ in the Bayesian learning model by a factor of 1.48 (that is $\sigma_\rho = 1.48$ JND). 9 participants (including one of the authors) volunteered for this experiment.

Results

The results for the shape discrimination task are shown in Figure S1C for an example participant. The separate colours indicate the results for the separate standards: red: $\rho = 0.1$, green: $\rho = 0.5$ and blue: $\rho = 0.9$. The points indicate the proportions of comparison shape perceived as less spiky for a given standard-comparison pair and the solid lines indicate the fitted psychometric curves. For the example participant the curves for the three different standards are very similar in terms of JND.

Figure S1D shows the median (bars) and 15% and 85% percentiles (errorbars) across observers. Individual participant results are plotted as separate points. A non-parametric Friedman test was performed on the JNDs to test for differences between the three standard shapes but no significant difference was found ($p = 0.12$). Therefore we can safely assume that the chosen shape-scale is relatively uniform across the different morph factors.

In the main experiment the minimum shape difference in morph factor between test shapes was 0.125. To verify whether these different test shapes would be distinguishable from each other we performed one-sided signed rank tests comparing the JNDs for each of the three standards to the minimal morph factor difference of 0.125. For each of the three standards the JNDs were significantly smaller than 0.125 ($p < 0.05$ after Bonferroni correction) indicating that the separate test shapes used in the main experiment are easily distinguishable from each other.

[1] The comparison -0.1 relative to the standard ρ of 0.1 was clamped to 0.01.

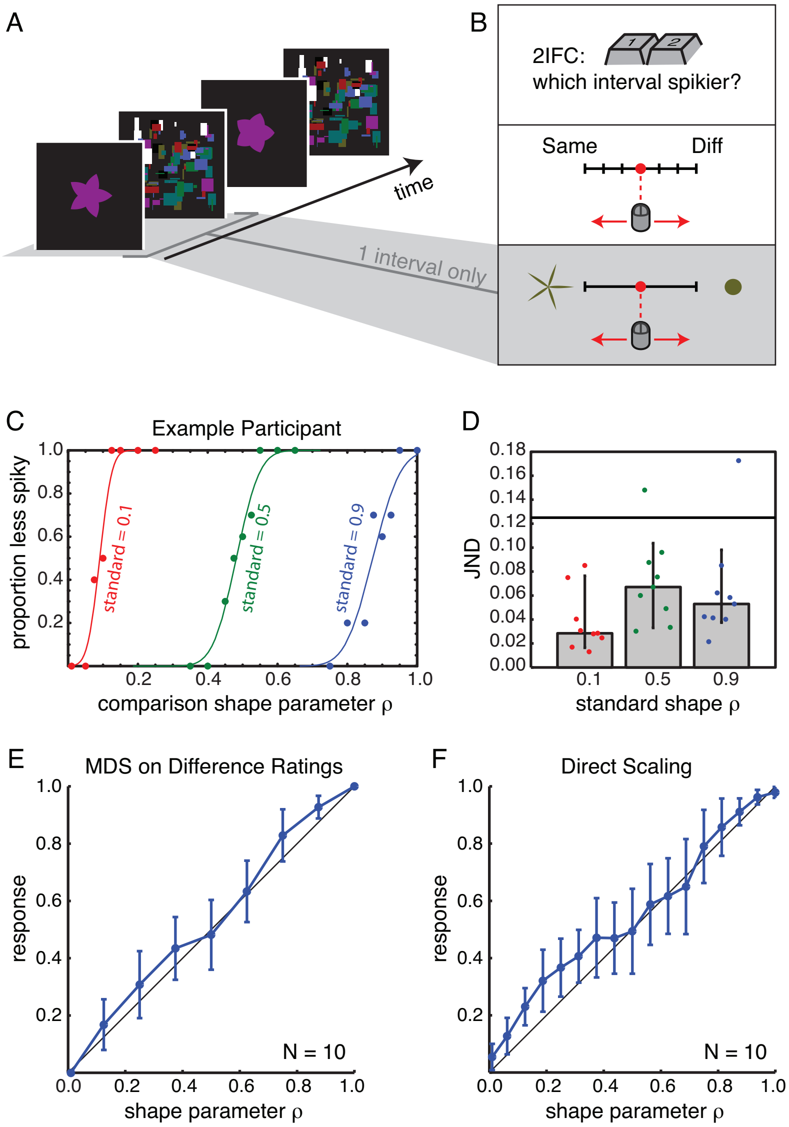

Figure S1: A) stimulus sequence for a single trial of in Control Experiment 1 (shape discrimination) and 2 (MDS-Scaling). For trials of Control Experiment 3 (Direct Scaling) the sequence consisted of only one shape followed by the mask. B) The different tasks used in the three Control Experiments. Top: for the JND measurements of Control Experiment 1, participants performed a 2-AFC task in which they had to respond which interval contained the most spiky shape. Middle: in Control Experiment 2, participants rated the difference between the two shown shapes to obtain dissimilarity ratings for use in the MDS analysis. Bottom: in Control Experiment 3, only a single shape was shown and participants directly placed the cursor where they thought the shape fitted between the two extreme shapes (Direct Scaling task). C) Example participant results for Control Experiment 1 showing the individual data points and psychometric fits for the three different standards. D) Results of Control Experiment 1 across participants. Bars indicate median JNDs and error bars the 15% and 85% percentiles across participants. Results for individual participants are shown as individual points in the plot. The continuous line at 0.125 indicates the smallest morph factor difference between the separate shapes in the main experiment. E) and F) Average results and standard deviations across participants for Control Experiment 2 (MDS-Scaling) and 3 (Direct Scaling), respectively. These graphs show the perceived scale as a function of the shape's ρ.

Control Experiment 2: Multi-Dimensional Scaling

On each trial of Control Experiment 2 two shapes were presented in the same fashion as for Control Experiment 1 (see Figure S1A). However, this time the participants' task was to rate the dissimilarity between the two shapes on a 7-point scale. To do so, a scale was presented visually (see Figure S1B, middle) and using a computer mouse participants could move a cursor to one of seven possible positions. The cursor always started in the middle of this 7-point scale. Participants were instructed to position the cursor on the leftmost mark when they thought the two presented shapes were exactly the same (zero dissimilarity), and otherwise to use one of the other six marks depending on the perceived dissimilarity: the more to the right, the more dissimilar the shapes. Furthermore, participants were encouraged to use the full scale of possible answers. Participants indicated by button press when they were satisfied about their rating.

All possible pairs of the 9 shape-morphs used in the main experiment (ρ is one of 0.01, 0.125, 0.25, 0.375, 0.5, 0.625, 0.75, 0.875, 1.0) were presented four times in counterbalanced order. This resulted in 180 trials (45 pairings $\times$ 4 repetitions) which were presented in random order. 10 naive participants volunteered for this control experiment.

Analysis

The MDS analysis was performed for each participant separately. As noted, the ratings from the participant were made on a 7-point scale, ranging from left (exactly the same shape) to right (very dissimilar). For each shape pair the four ratings for a participant were averaged to obtain the perceived dissimilarity for that pair. These averaged dissimilarity ratings were then used to perform the non-metric MDS along a single dimension ("MDScale" in MATLAB). The average MDS stress across participants using one dimension was found to be 0.059 which indicates a reasonably good Goodness of fit [1].

Since non-metric MDS only provides the relative relationship between the different shapes but not the absolute scale or orientation of this relationship, we reoriented the MDS results using linear transformations. These transformation were performed such that for the two extreme shapes ($\rho = 0.01$ and $\rho = 1.0$) the perceived "ρ" matched the physical ρ. All the other shapes were spaced relative to these extreme shapes according to the MDS relative spacing results. This way, the mapping from the ρ-shape scale to the perceived shape scale was obtained for the individual participants. After this realignment the mapping from physical to perceived shape scales were averages across participants.

Results

The results for Control Experiment 2 are shown in Figure S1E. As can be seen the perceived scaling of the shapes versus the shape-scale in terms of ρ is indistinguishable from linear ((Ramsey Reset test [2] with k = 3: p = 0.62).

Control Experiment 3: Direct perceptual scaling

In Control Experiment 3, we used a more direct approach to obtain a second measure for the relationship between physical ρ and the perceived spacing between the shapes. Participants were shown only a single shape for 1 sec, followed by the multi-rectangle mask for 1 sec. Next, a scale appeared indicating the most spiky shape on the left ($\rho = 0.01$) and a perfect circle on the right ($\rho = 1.0$). A red dot functioned as a cursor which participants could move in a continuous manner along the range of the scale by moving a computer mouse. The participants task was to position the cursor at the location on the scale where they felt the single shape fitted between the two extreme shapes (Figure S1B, bottom). Again, for each trial the cursor always started in the middle of the scale so as not to bias the participants in their individual responses. Participants indicated by button press when they were satisfied with their response.

Participants performed 10 repetitions for 17 shapes in total (i.e., 170 trials). The 17 shapes used were equally spaced in steps of $\Delta\rho = 0.0625$ between the two extreme shapes along the ρ-scale. For each participant the results for the 10 repetitions for each shape were averaged to obtain the perceived position of the shape along the ρ-scale. The same 10 participants as for Control Experiment 2 volunteered for this task. The order of the two experiments (Control Experiment 2 and 3) was counterbalanced across participants.

Results

The average results for Control Experiment 3 (Direct Scaling) are shown in Figure S1F. Again, any deviations from linearity are small, such that the results are indistinguishable from linear (Ramsey Reset test with k = 3: p = 0.094).

Since the same participants performed both Control Experiment 2 and 3, we investigated the within participant consistency of the responses in the two experiments. On average the correlation between the results of two experiments was 0.99 for the direct comparison of perceived scale and 0.67 for the perceived

	Main text (ρ shape scale)		Control Experiment 2 (MDS Scaling)		Control Experiment 3 (Direct Scaling)	
Model	MKF	Linear	MKF	Linear	MKF	Linear
2 pairs (Exp 1)	0.77	0.60	0.77	0.60	0.77	0.60
2 pairs (Exp 2)	0.85	0.44	0.85	0.44	0.86	0.38
5 pairs (Exp 2)	0.65	0.27	0.65	0.27	0.67	0.25
3 pairs (Exp 3)	0.85	0.57	0.86	0.60	0.86	0.58

Table S1: R^2 for the Mixture-of-Kalman-Filters (MKF) model and the linear rule learning prediction. From left to right results are shown for a linearly perceived shape scale in terms of ρ as used in the main text, the perceived shape scale as found using the MDS method in Control Experiment 2 and the perceived shape scale as found in Control Experiment 3 using the Direct Scaling method.

differences between consecutive shapes for the shapes that were used in both paradigms. This means that the results from Control Experiment 3 were very similar to those of Control Experiment 2 even on an individual participant basis.

Model verification

The results of all three Control Experiments show that the perceived shape scale is indistinguishable from linear when expressed in terms of ρ. Particularly, the results of Control Experiments 2 and 3 show that deviations from a linear relationship are generally very small. However, the visible trend of small deviations seem very consistent across the two control experiments (Figure S1E and F). To analyse if any slight deviations could help further explain the results of the shape-mapping association learning experiments described in the main text, we used the perceived scale as obtained from both Control Experiment 2 and 3 to make new predictions based on the linear rule learning prediction and using the Mixture-of-Kalman filters model. That is, we applied both the linear rule learning hypothesis and the Mixture-of-Kalman filters model, on the perceived shape scale as obtained from Control Experiment 2 and 3 instead of on the ρ-scale directly. The predictions obtained in this way were then transformed, using the relationship shown in Figure S1E and F, respectively, to obtain the predictions in terms of the ρ-shape scale. We again calculated the R^2-goodness-of-fits for these predictions with respect to the shape generalisation results shown in the main text.

In Table S1 the average R^2-goodness-of-fits are shown for this analysis. For a better comparison the R^2 as mentioned in the main text are presented in the first two columns, for the Mixture-of-Kalman filters model and the linear rule learning hypothesis respectively. From Table S1 it is evident that, since

deviations in linearity between the perceived shape scale and the mathematical ρ-shape scale were only small, this analysis only led to very slight changes in R^2. This further supports our assumption that the shape-scale in terms of ρ is perceptually linear and small deviations do not change the overall conclusions.

References

1. Kruskal J (1964) Multidimensional-scaling by optimizing goodness of fit to a nonmetric hypothesis. Psychometrika 29: 1-27.

2. Ramsey J (1969) Tests for specification errors in classical linear least squares regression analysis. Journal of the Royal Statistical Society B 31: 350–371.
