# Supplementary material for: Mapping Shape to Visuomotor Mapping: Learning and Generalisation of Sensorimotor Behaviour Based on Contextual Information
Source: PLoS Comput Biol. 2015 Mar 27;11(3):e1004172. doi: 10.1371/journal.pcbi.1004172 (PMC4376781; doi:10.1371/journal.pcbi.1004172)
Supplement: S3 Text — (PDF) [file pcbi.1004172.s003.pdf]

S3 Intermanual transfer of learning

To investigate the extent to which learning the shape-mapping associations can be considered implicit or explicit we examined the amount of intermanual transfer of the learned associations between shape and response. If learning is fully explicit it should be straightforward to apply the simple associations to both the trained as well as the untrained hand. If, on the other hand, learning is largely implicit the learning likely is dependent on the hand used and thus intermanual transfer should hardly occur. Note, however, that even for very straightforward prism and visuomotor feedback rotation adaptation paradigms, for which learning is considered to be mostly implicit, intermanual transfer does generally occur, but only to a very limited extent.

We compared two conditions: the first 2-Pair Hand-Transfer Condition was very similar to Experiment 1 of the main text, i.e., the same shape mapping pairs were trained. For the second condition, participants were trained on a single shape-mapping association only ($\rho = 0.5$, mapping = 75mm). In this case, learning is consistent with adaptation to a single visuomotor shift which, as noted above, is generally considered to be mostly implicit. Thus, this 1-Pair Hand-Transfer Condition serves as the baseline for this control experiment.

17 right-handed participants volunteered (9 and 8 for the 2-Pair and 1-Pair Hand-Transfer Conditions respectively). The results for one participant in the 2-Pair Hand-Transfer Condition were removed since he failed to learn the shape-mapping associations. For both conditions, half the participants used their dominant right hand during training and the other half their non-dominant left hand.

The procedure was the same as for the experiments in the main text. The only difference was that catch trials were not randomly interleaved between training trials. Instead short periods of 9 catch trials each (1 trial for each generalisation shape) were interleaved at regular intervals between the training trials in the last two blocks of the session. These catch-trial periods were performed in an alternate fashion for the trained hand and the untrained hand and for each hand there were ten such catch-trial periods. Participants were notified which hand to use for each catch trial period by a message on the screen. To ensure that the participants did not simply continue without taking notice of the message, they additionally needed to press a button on the graphics tablet in order to proceed with the experiment. Note, that since generalisation was measured for both the trained and the untrained hand, the number of catch trials in the procedure was doubled compared to the experiments described in the main text.

To accommodate the increased number of catch trials, without changing the training paradigm itself, we therefore added an additional block of trials to the paradigm. That is, for each participant the experiment consisted of 6 experimental blocks, the first 4 consisted of 180 training trials each and the last two contained both additional training trials as well as the catch-trial periods.

Results

The results of the intermanual transfer experiment are shown in Figure S2. For the 2-Pair Hand-Transfer Condition there is a substantial amount of transfer between the hands (Figures S2A and B). When only the trained shape-mapping pairs are considered (Figure S2B) there is no significant difference between the results for training trials, catch trials for the trained hand, and catch trials for the untrained hand (pairwise paired t-tests $p > 0.05$). A 2-way (shape $\times$ hand) repeated measures ANOVA on all the catch trials results (that is, also catch trials for shapes other than the trained shapes) did however show a significant interaction between shape and hand ($p = 0.04$) indicating a slight decrease in the overall learning and generalisation trends for the untrained hand (see also Figure S2A).

In contrast, the results for the 1-Pair Hand-Transfer Condition showed only a small amount of transfer from the trained to the untrained hand (Figure S2C and D). For the trained shapes the differences between training trials, catch trials for the trained hand and catch trials for the untrained hand were all significant (pairwise paired t-tests $p < 0.01$, see Figure S2D). Furthermore, the 2-way repeated measures ANOVA with catch trial shape and hand as independent variables showed a significant main effect for the hand used ($p = 0.003$).

In short, transfer of learning between the hands was quite substantial for the 2-Pair Hand-Transfer Condition compared to the 1-Pair Hand-Transfer Condition. Together with the results that learning only occurred when participants were aware of the role of the shape, this indicates that learning the shape-mapping associations was mostly explicit.

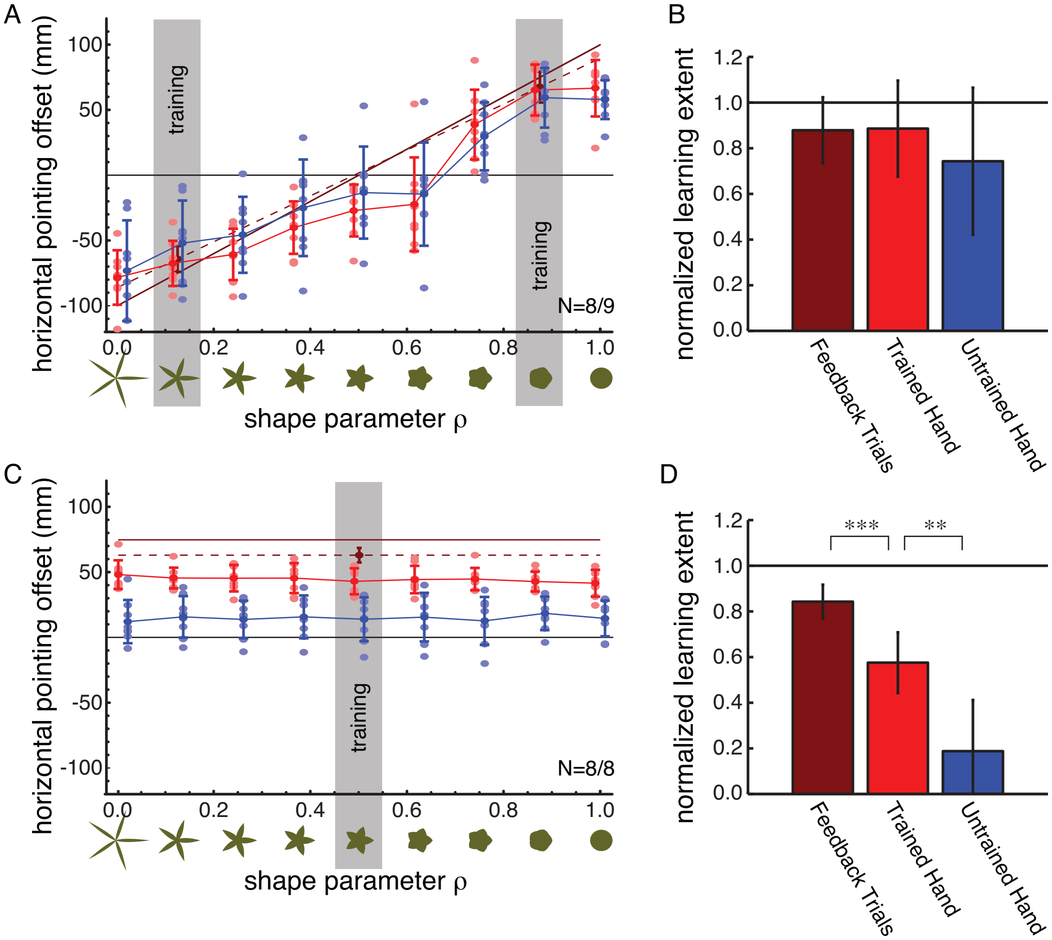

Figure S2: Results for the intermanual transfer control experiment. A, C) show the generalisation results for the 2-Pair Hand-Transfer Condition and 1-Pair Hand-Transfer Condition respectively. Dark red dots and error bars show the results for the training trials in the last two experimental blocks. Light red symbols and lines show the results for catch trials in which the trained hand was used. Blue symbols and lines show the results for catch trials in which the untrained hand was used. C, D) show the normalised learning extent for the trained shape-mapping pairs for the 2-Pair and 1-Pair Hand-Transfer Conditions, respectively.
